# Neurobiological Responses towards Stimuli Depicting Aggressive Interactions in Delinquent Young Adults and Controls: No Relation to Reactive and Proactive Aggression

**DOI:** 10.3390/brainsci12020124

**Published:** 2022-01-18

**Authors:** Janna F. ter Harmsel, Josanne D. M. van Dongen, Josjan Zijlmans, Thimo M. van der Pol, Reshmi Marhe, Arne Popma

**Affiliations:** 1 Department of Child and Adolescent Psychiatry, Amsterdam UMC, Vrije Universiteit Amsterdam, 1105 AZ Amsterdam, The Netherlands; j.zijlmans@amsterdamumc.nl (J.Z.); thimo.vander.pol@inforsa.nl (T.M.v.d.P.); marhe@essb.eur.nl (R.M.); a.popma@amsterdamumc.nl (A.P.); 2 Forensic Mental Health Care, Inforsa, 1059 GL Amsterdam, The Netherlands; 3 Department of Psychology, Education & Child Studies, Erasmus University Rotterdam, 3062 PA Rotterdam, The Netherlands; 4 Department of Research and Quality of Care, ARKIN Mental Health Institute, 1033 NN Amsterdam, The Netherlands

**Keywords:** aggressive behavior, psychophysiology, electrophysiology, reactivity, emotion regulation, young adulthood

## Abstract

Neurobiological measures underlying aggressive behavior have gained attention due to their potential to inform risk assessment and treatment interventions. Aberrations in responsivity of the autonomic nervous system and electrophysiological responses to arousal-inducing stimuli have been related to emotional dysregulation and aggressive behavior. However, studies have often been performed in community samples, using tasks that induce arousal but not specifically depict aggression. In this study, we examined differences in psychophysiological (i.e., heart rate, respiratory sinus arrhythmia, skin conductance level) and electrophysiological responses (i.e., P3, late positive potential, mu suppression) to aggressive versus neutral scenes in a sample of 118 delinquent young adults and 25 controls (all male, aged 18–27). With respect to group differences, we only found significant higher SCL reactivity during the task in the delinquent group compared to controls, but this was irrespective of condition (aggressive and neutral interactions). Within the delinquent group, we also examined associations between the neurobiological measures and reactive and proactive aggression. No significant associations were found. Therefore, although we found some indication of emotional dysregulation in these delinquent young adults, future studies should further elucidate the neurobiological mechanisms underlying emotional dysregulation in relation to different types of aggression.

## 1. Introduction

Aggressive and violent behavior in young adulthood is a major concern in forensic psychiatry and society, given the negative impact of concomitant social problems and delinquency on victims and perpetrators, as well as the high costs for health care and society [1,2]. Although treatment-associated risk reductions in violent recidivism have been reported in several studies [3], the current overall efficacy of psychological and psychosocial interventions aimed at reducing aggressive behavior in forensic patients is found to be limited [4,5].

Over the last years, studies focusing on neurobiological factors of aggression have increased (for an overview, see [6]), aiming to improve risk assessment and treatment of aggressive behavior [7]. One of the underlying mechanisms that is found to be disrupted in aggressive behavior is emotion regulation [8,9]. Studies on the neurobiology of emotion regulation in delinquents have mostly examined either structural or functional brain correlates or used psychophysiological or electrophysiological measures. Thus, neurobiological measures are rarely jointly studied in relation to aggression [10]. In addition, studies often have focused on antisocial behavior more generally, leaving aggressive behavior less specifically addressed. For instance, a recent review of neuroimaging of psychopathic traits [11] showed structural and functional impairments in psychopathy, antisocial personality disorder and conduct disorder to be related to frontotemporal, limbic, paralimbic and cerebellar regions. However, in that review, no studies on aggression in general, nor to reactive or proactive aggression more specific were discussed. In the current study, we therefore examined both psychophysiological and electrophysiological responses towards aggressive scenes in delinquent young adults and controls and examined associations between these neurobiological measures and two different types of aggressive behavior.

Aggressive behavior can be classified in multiple ways, for example according to expression (physical vs. verbal aggression) or nature (direct vs. indirect aggression). In treatment of aggression, clarifying the intentions to engage in aggressive behavior is highly relevant. The most used differentiation in aggressive behavior, reactive vs. proactive aggression, is based on this motivational aspect [12]. Whereas reactive aggression is defined as an impulsive response to a provocation or threat, rooted in the frustration-aggression theory [13], proactive aggression is characterized by instrumental, premeditated behavior to achieve a secondary goal, explained by social learning theory [14], fearlessness theory [15] or sensation-seeking theory [16]. Although these two dimensions of aggressive behavior, as currently operationalized in self-report questionnaires, are highly correlated [17,18,19], several studies have demonstrated unique predictors for both constructs. For example, researchers have found that although antisocial behavior is related to both reactive and proactive aggression, reactive aggression is uniquely related to a hostile interpretation bias [20]. Moreover, impulsivity and self-control are found to be uniquely related to reactive aggression [21], while amongst others, psychopathic personality [18,22] and lower attentional bias towards aggressive words [23] are uniquely predictive of proactive aggression. These findings suggest that reactive aggression and proactive aggression may arise from distinct underlying (neurobiological) mechanisms.

Psychophysiology is a collective term for all neurobiological measures related to the activity of the autonomic nervous system (ANS), the human regulatory system which regulates respiration, heartbeat and sweat secretion. Whereas some measures provide an indication of general ANS functioning, other measures are indicative of activity of one of the branches of the ANS: the sympathetic nervous system (SNS; the ‘accelerator’) or parasympathetic nervous system (PNS; the ‘brake’), which usually cooperate in a reciprocal way. Furthermore, measures can be taken at rest or in response to arousal-inducing events (i.e., reactivity measures), such as exposure to social stressors, provocation or emotion-eliciting pictures or film clips. In aggression research, lower heart rate (HR) at rest has most consistently been found to be positively associated with antisocial behavior in general [2] and proactive aggression, although the overall effect size is found to be small [24]. However, the findings regarding reactivity of the ANS, SNS and PNS, which is the focus of our study, are less robust.

Considering general ANS reactivity, with HR as indicator, meta-studies have shown increased HR during stressor tasks among antisocial youth see [25] for a meta-analysis and small positive associations between aggression and HR reactivity to emotional stimuli with a negative valence [26]. Furthermore, increased HR reactivity to provocation is found to be associated with reactive violence, but also with nonviolent delinquency [27]. However, other studies did not support these findings [28,29]. Proactive aggression is found to be uniquely associated with blunted HR activity [29], but see [30], for contradictory results.

Research results regarding SNS reactivity, indicated by skin conductance level (SCL), are mixed as well. Reactive aggression is found to be associated with heightened SNS reactivity in response to provocation [31,32,33,34] and fear [35]. In contrast, proactive aggression has been linked to blunted skin conductance reactivity [31,35,36], although no association has also been reported [28,37].

Research findings for the PNS, with respiratory sinus arrhythmia (RSA) as indicator, are equivocal as well. Increased RSA withdrawal in anger-inducing situations has been linked to externalizing symptoms [38] and self-regulation deficits in antisocial youths [39]. With regard to reactive aggression, RSA withdrawal is found to be positively associated with reactive aggression [40], although no associations have been reported as well [28,37]. Proactive aggression is more consistently found to be related to blunted PNS reactivity [31,37,41].

Given these mixed reactivity outcomes, the support for theoretical models stating that reactive aggression is associated with heightened arousal or hyper-reactivity of the ANS and that proactive aggression is related to low arousal or hypo-reactivity of the ANS [29,42], is limited. Furthermore, in recent research the association between aggressive behavior, emotion regulation and the interaction of the two branches of the ANS is highlighted [29,43]. According to this view, reactive aggression results from coactivation of the SNS and PNS, while proactive aggressive might stem from co-inhibition of both systems [36,44,45]. Building on this, researchers suggest that aggressive behavior can be better predicted by the interaction between SNS and PNS than by hypo- or hyperreactivity of each of these subsystems alone [29,36].

Electroencephalography (i.e., EEG) is an electrophysiological measure that assesses electrical activity via electrodes at the scalp level. Common EEG indices are event-related potentials (i.e., ERPs) and frequency power. ERPs are responses to particular events (i.e., stimuli) and are time locked to that event. For instance, two well studied ERPs related to emotion regulation and arousal are the P3 and late positive potential (LPP) between 300 and 700 ms post-stimulus over centro-parietal regions [46,47]. These ERPs are found to be associated with directed attention and evaluation of stimuli and are also found to be modulated by emotion. This means that when emotional arousal levels rise, the amplitudes of these ERPs also increase. Therefore, P3 and LPP in affective picture processing tasks are often used as correlates of emotional arousal and emotion regulation indices [48].

Generally, attenuated P3 amplitudes are found to be related to externalizing behavior, including antisocial behavior and aggression [49,50] indicating the ineffective processing of salient affective stimuli. It has been demonstrated that within the normal population, individuals who self-reported as being reactive aggressive, presented with significantly lower P3 amplitude in frontal electrode sites compared with non-aggressive controls [51]. In contrast, proactive aggressors are found not to show this reduced P3 component characteristic of impulsively aggressive individuals [52,53]. However, it has also been found that both reactive and proactive aggression are associated with attenuated P3 amplitudes [54].

With respect to the LPP, findings are also mixed. While in one study, it was found that higher aggressive individuals showed an attenuated LPP in reaction to violent pictures [55], and that this attenuation was linked to impulsivity scores in that group. Another study [56], found an increased LPP was associated with impulsive (reactive) aggression in aggressive individuals, indicating more arousal towards these as hostile interpreted situations.

Assessing event-related brain perturbations in terms of its frequency characteristics can provide unique insights into emotional stimulus processing. Specifically, event-related oscillations not only assess stimulus-evoked oscillations akin to the traditional ERP analysis but also induced oscillations, which are not phase locked to the stimulus event. Frequency power is the oscillatory activity measured in a particular frequency band (i.e., delta, theta, alpha, beta and gamma), and activity in each of these frequency bands have been found to be related to particular cognitive characteristics. For instance, theta-band (4–8) oscillations have been found to be associated with memory and cognitive control [57], while alpha power is generally associated with spatial attention [58].

Several studies assessed event-related oscillations associated with emotional stimulus processing. However, findings are difficult to integrate because studies focus on different frequencies, time windows, topographies and variations in task instructions. However, generally the processing of emotionally arousing (pleasant and unpleasant) compared to neutral stimuli (i.e., words; [59], facial expressions [60,61,62,63,64] and images from the International Affective Picture System (IAPS) [65,66,67] are often associated with a decrease in alpha power (i.e., event-related desynchronization; ERD). This indicates that alpha ERD is related to emotional arousal [48,68].

With respect to aggression, previous studies using resting-state EEG found decreased alpha power in centro-parietal regions to be related to psychopathic personality in violent offenders [69]. A more recent study showed that alpha ERD at frontal sites was related to aggression and retaliation [70], providing support for the idea that aggression results from a decrease in self-control, including dysfunctional emotion regulation.

Interestingly to add, both the LPP and alpha ERD have been found to be similarly modulated by affective arousal [66], pointing to the relevance to study those two correlates conjointly. Interestingly, recent findings show alpha desynchronization specifically at central-parietal electrodes (which is sometimes referred to as mu suppression) in the 500–1000 ms time range to be associated with empathy during vicarious pain [71]. This time range is consistent with the time window in which the LPP is typically found.

In sum, research into the neurobiological mechanisms underlying aggressive behavior has gained attention over the last years due to their potential to inform risk assessment and development of tailored treatment interventions to reduce aggressive behavior. However, studies are often performed in community samples, using resting-state data or neuropsychological tasks that induce arousal but not specifically depict aggressive behavior. Furthermore, results remain inconsistent and neurobiological measures are rarely studied conjointly [10]. Therefore, the aim of our study was to examine differences in psychophysiological (i.e., HR, RSA, SCL) and electrophysiological responses (i.e., P3, LPP, mu suppression) between delinquent young adults and controls in a passive viewing task depicting aggressive and neutral interactions. Within the delinquent group, we also examined associations between these neurobiological measures and reactive and proactive aggression.

In line with the literature, we hypothesized emotion dysregulation to be present in the delinquent group, but not in the control group. Given the mixed findings in previous research, we expected altered reactivity of the ANS measures in the delinquent group compared to the control group. For the electrophysiological measures, we expected decreased ERP amplitudes and less alpha ERD/mu suppression in the delinquent group compared to the control group. Furthermore, for the psychophysiological reactivity measures, we expected positive associations with reactive aggression (primarily SCL and HR) and negative associations with proactive aggression (primarily RSA). In addition, electrophysiological reactivity measures were expected to be specifically negatively related to reactive aggression.

## 2. Materials and Methods

### 2.1. Participants

In total, 154 young adult men participated in the neurobiological part of a larger study [72]. In 11 participants, both psychophysiological and electrophysiological data were not properly recorded, stored, or otherwise missing. We therefore studied a group of 143 young adults, all male, aged 18–27 years (M = 22.60, SD = 2.43). The experimental group (*N* = 118) consisted of young adults with social, psychological and addiction problems, almost all with a history of delinquent behavior, following a multimodal day-treatment program at De Nieuwe Kans, a day-treatment center in Rotterdam, The Netherlands [73]. This program employs cognitive behavioral techniques and rehabilitation components, such as cognitive skills training, drug treatment and education [72]. The participants were recruited at the start of their treatment program at De Nieuwe Kans. Age-matched controls (*N* = 25) were recruited at colleges for intermediate vocational training in Rotterdam and selected to have education levels that matched the delinquent sample. Demographic information for each group is displayed in Table 1. All participants provided written informed consent. The study was conducted according to the guidelines of the Declaration of Helsinki and approved by the Medical Ethics Committee of the VU University Medical Center (registration number 2013.422–NL46906.029.13). Participants received 30 euros for participation in this part of the study.

### 2.2. Materials

#### 2.2.1. Questionnaires

For this study, two questionnaires were selected from a larger test battery. A self-developed questionnaire was used to assess demographic characteristics (age, gender, ethnicity, education level and history of delinquent behavior). To assess the two differently motivated types of aggressive behavior, the Reactive Proactive Questionnaire (RPQ) [17], a 23-item self-report measure, was administered. The RPQ uses a 3-point Likert scale (0 = never, 1 = sometimes, 2 = often) to assess physically and verbally aggressive behaviors as well as anger in response to external stimuli. The two-factor structure of the original RPQ was also found for the Dutch version. Convergent validity of this version was reported as adequate (all *r*’s > 0.16) and test–retest stability as good (all ICC’s > 0.41) in a validation study [74].

#### 2.2.2. Passive Picture Viewing Task

**Figure 1:**
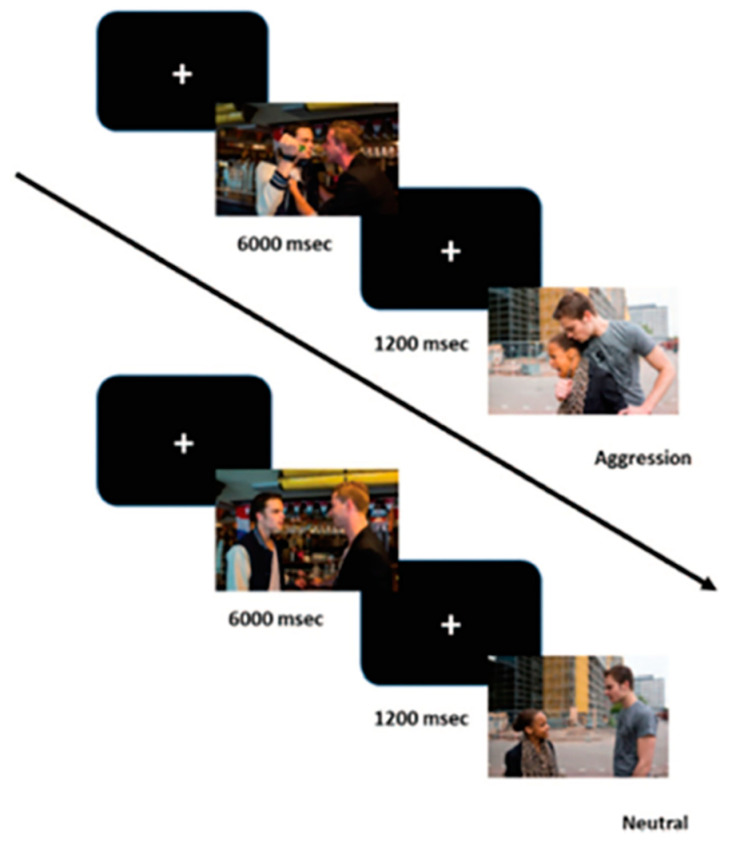
Passive viewing task with pictures of aggressive interactions and neutral interactions serving as control pictures.

To induce arousal, a passive viewing task with pictures of aggressive and neutral interactions was used (see Figure 1). This task was originally developed to assess empathic processing and is described in greater detail by the developers [75]. The task consists of 40 picture pairs, with either two male protagonists or one male and one female, all between 20 and 25 years of age. The male actors had a white complexion, and the female had a black skin tone. In all but one of the aggression scenes, in which physical, sexual, and verbal aggression were depicted, a male individual was the perpetrator. All neutral pictures were pairwise matched to the aggressive pictures, using the same persons, location, colors and light. Three types of pictures were used in our experiment: (a) 40 pictures depicting a violent interaction between two individuals; (b) 40 pictures depicting a neutral interaction between two individuals; and (c) 15 pictures depicting neutral objects (i.e., filler items), which were not used for further analyses. A total of 95 pictures were randomly presented for 6 s with intervals of 1.8 s between the pictures. All pictures were presented in full-screen mode on a color monitor located at approximately eye level about 1.5 m in front of the participants. Participants were instructed to passively look at each picture.

#### 2.2.3. Psychophysiological Measures

Psychophysiological measures were collected at the Erasmus Behavioral Laboratory of the Institute for Psychology at the Erasmus University Rotterdam, using the VU Ambulatory Monitoring System (VU-AMS, Amsterdam, The Netherlands) [76]. During the neuropsychological test-paradigm, participants were seated in a comfortable chair in a sound-attenuated room with dimmed lights. A sampling rate of 1000 Hz was used. Placement of the five ECG Micropore electrodes (Kendall H98SG) for electrocardiography (ECG) and impedance cardiography (ICG) was carried out according to the VU-AMS manual (http://www.vu-ams.nl/support/instruction-manual/, last accessed on 24 November 2021). Two skin conductance electrodes (Biopac TSD203) were placed on the medial phalanges of the middle and index finger of the non-dominant hand, using isotonic electrode gel (4 OZ, GEL101). Participants were instructed to move as little as possible and not to touch the electrodes.

Data preparation was performed following the instructions in the VU-AMS manual. In this study, we focused on HR, RSA and SCL during the passing viewing task. HR and RSA were both derived from ECG and ICG measures by the VU-AMS system. HR was assessed by automated counting of R peaks (beats per minute) and RSA was calculated by distracting the shortest period between heart beats during inspiration from by the longest period between heart beats during expiration. SCL (in microSiemens) during the task was automatically registered by the VU-AMS system.

Data processing, including R-peak time series analysis, took place using automated detection algorithms of the VU-AMS Data, Analysis and Management Software (VU-DAMS, Amsterdam, The Netherlands). Suspected Inter Beat Intervals (IBI’s), relevant for both HR- and RSA-analysis, were manually checked for artefacts as well as missing or incorrect R-peaks and adjusted if necessary. SCL-data were checked for artefacts and values outside the range recommended in the VU-AMS manual (<1 and >12). Extreme outliers, emerging from outlier analysis, were removed.

#### 2.2.4. Electrophysiological Measures

Brain activity during the task was recorded with EEG using a Biosemi ActiveTwo System amplifier (Biosemi, Amsterdam, The Netherlands) and active Ag/AgCl electrodes at 32 standard (10–20 International System) scalp sites and two additional scalp sites (FCz and CPz). Four additional electrodes were used to measure vertical and horizontal electro-oculogram. They were placed above and below the left eye and at the outer canthi of the eyes, respectively. Two other additional electrodes were placed on the left and right mastoids. All signals were digitized with a sampling rate of 512 Hz and 24-bit analog-to-digital conversion and were filtered offline.

Data were processed offline using the Brain Vision Analyzer software (BVA, Brain Products, Gilching, Germany). First, the EEG signal was re-referenced offline to the linked mastoids. Because we were interested in both time (P3 and LPP) and frequency (alpha ERD) properties, segmentation was done per condition (aggression vs. neutral) in an interval between −200 and 1500 ms (ERPs) and −1000 and 3000 ms (alpha power) relative to stimulus presentation. Subsequently, data were filtered using a bandpass filter ranging from 0.01 Hz to 30Hz (phase shift-free Butterworth filters; 24 dB/octave slope, and the Gratton and Coles algorithm [77] was used to correct for eye movements and blinks.

**Figure 2:**
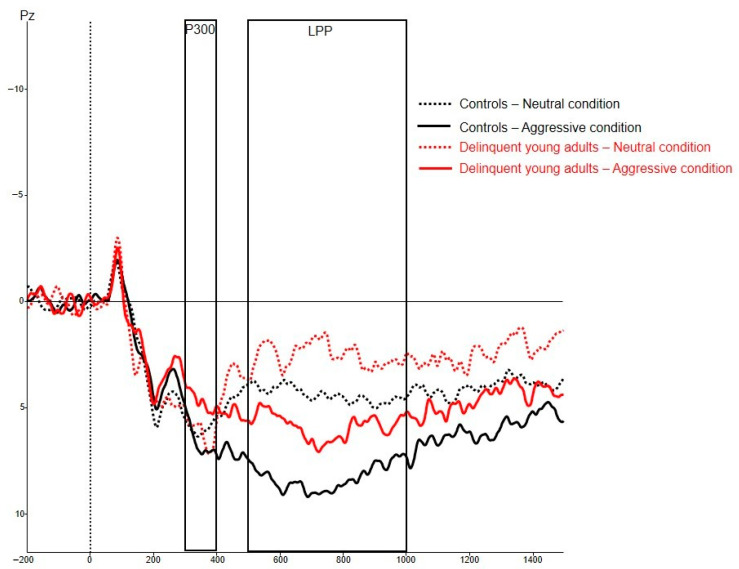
Grand average ERPs at Pz.

For the ERPs, a baseline correction was performed using a 200 ms pre-stimulus interval, and remaining artifacts (i.e., segments with an EEG signal exceeding an amplitude of 75 V) were removed. Visual inspection of the grand average ERPs at Pz (see Figure 2) revealed a clear positive wave, which was maximal for aggressive pictures between 300–400 ms after stimulus onset (i.e., the P3) and was followed by sustained slow wave activity (i.e., the LPP). For ERP analyses, the P3 amplitude was defined as the mean amplitude at Pz between 300 ms and 400 ms after stimulus onset [78]. The LPP was defined by the mean amplitude at Pz between 500 ms and 1000 ms after stimulus onset [79]. The average signals per condition were then used for determining ERP characteristics.

As we were also interested in the oscillations underlying the processing of these aggression pictures, we used time-frequency analysis to study the total alpha power at central sites (i.e., mu power) using Morlet wavelets. Because the processing of emotions and social interactions (including empathic processing) is most prominent in the right hemisphere [80,81], electrode site C4 was used for determining alpha ERD (i.e., mu suppression). After segmentation, ocular correction and filtering, and remaining artifacts (i.e., segments with an EEG signal exceeding an amplitude of 150V) were removed. After preprocessing, we performed a wavelet analysis in the 1- to 80-Hz frequency range to obtain power values for the EEG activity at different time points for each segment and for each condition. We used a complex Gaussian Morlet wavelet (Morlet parameter set to 5) and 60 logarithmic frequency steps and averaged the obtained power values for each time-frequency point. Edge and smearing effects were accounted for by reducing the window of baselining and analyses, respectively, with half the wavelength [82]. After averaging the total power per condition, we extracted the layer that best fitted the alpha band (between 8 Hz and 13 Hz). During data processing, data of any participant having fewer than 20 clean segments (i.e., half of the maximum number) per condition after artifact rejection were excluded from the analyses.

#### 2.2.5. Data-Analysis

Neurobiological responses during the task and self-reported aggressive behavior were statistically evaluated using IBM SPSS 27 and R 4.0.2. First, we performed a descriptive and comparative analysis using Welch’s tests (assuming unequal variances given the difference in sample size between both groups) on the behavioral and neurobiological measures. Second, to account for missing values (47.6% of the cases had missing data on one or more independent variables), multiple imputation was applied for predictors with a maximum missingness of 30%, creating 50 complete sets [83]. No data for dependent variables were imputed. Prior to imputation, Little’s Missing Completely At Random (MCAR) test was employed; the data were MCAR (χ2 = 69.730, df = 199, *p* = 1.000). Datasets were imputed using Multivariate Imputation by Chained Equations (MICE) in R [84]. Third, predictor variables were tested for normality of distribution, homoscedasticity, linearity and multicollinearity. Residuals were normally and equally distributed and linearity was not violated. Multicollinearity was tested by inspecting Variance Inflation Factors (VIFs). None of the VIFs exceeded 10, indicating no dependency between the variables [85]. Fourth, before running the main analyses, we performed pooled paired-sample t-tests over the entire sample to assess whether the task elicited expected neurobiological reactions during passive viewing of aggressive and neutral scenes. Fifth, we examined differences between delinquent young adults and controls in their neurobiological responses to aggressive and neutral scenes using six pooled Repeated Measures ANOVA’s using a 2 (condition; aggression vs. neutral) by 2 (group; delinquent vs. control) design. Sixth, we further investigated whether and how the neurobiological responses were associated with reactive and proactive aggression within the delinquent young adult group. In this group, reactive and proactive aggression were moderately correlated (*r* = 0.523, *p* < 0.001). Since we were interested in the unique explanation of reactive and proactive aggressive behavior, we performed two independent regression analyses. Two different regression analyses with pooled imputed data were performed, using the difference scores of the six neurobiological measures (aggression-neutral condition) as predictor variables. Cohen’s rule of thumb was used to assess effect sizes (Cohen’s d) with 0.01 being small, 0.09 being moderate, and 0.25 being large [86]. An alpha of 0.05 was used.

## 3. Results

### 3.1. Descriptive Analysis

After data processing psychophysiological measures of 118 participants and electrophysiological measures of 129 participants were eligible for further analysis. Descriptive statistics and first comparative results of the behavioral and neurobiological measures (original, non-imputed data) are presented in Table 2.

### 3.2. Task Validity

After data imputation, pooled paired sample t-tests revealed one significant neurobiological change in response to aggressive versus neutral interactions. LPP amplitude was significantly higher in response to aggressive behaviors compared to neutral behaviors (M = 2.80, *t* = 7.140, df = 171.19, *p* < 0.001). No significant differences between the two conditions were found for HR (*p* = 0.119), SCL (*p* = 0.776), RSA (*p* = 0.451), P3 (*p* = 0.725) and alpha power (*p* = 0.662).

### 3.3. Group Differences

#### 3.3.1. Psychophysiological Measures

Pooled Repeated Measures ANOVAs revealed no significant main effect for HR on condition (*p* = 0.159) nor group (*p* = 0.999). No interaction effect between condition and group (*p* = 0.992) was found.

Analyses of RSA also showed no significant main effect for condition (*p* = 0.345), nor group (*p* = 0.729). No interaction effect between condition and group (*p* = 0.518) was found.

Furthermore, SCL-analyses showed no significant main effect for condition (*p* = 0.816). However, the main effect for group was significant (F1,114 = −2.142, *p* = 0.034, *d* = 0.322), indicating that the delinquent young adults showed higher electrodermal activity compared to the controls, in response to both aggressive and neutral pictures. The interaction effect between group and condition was not significant (*p* = 0.946).

#### 3.3.2. Electrophysiological Measures

Pooled Repeated Measures ANOVAs showed no significant main effect for P3 amplitude on condition (*p* = 0.451) nor a main effect for group (*p* = 0.457). No interaction effect between condition and group was found (*p* = 0.447).

Analyses of the LPP amplitude resulted in a significant main effect for condition (F1,214 = 6.376, *p* < 0.001, *d* = 0.422), where aggression pictures led to higher LPP amplitudes compared with the neutral pictures in both the groups. No main effect for group was detected (*p* = 0.910). The interaction between condition and group was also not significant (*p* = 0.258).

**Figure 3:**
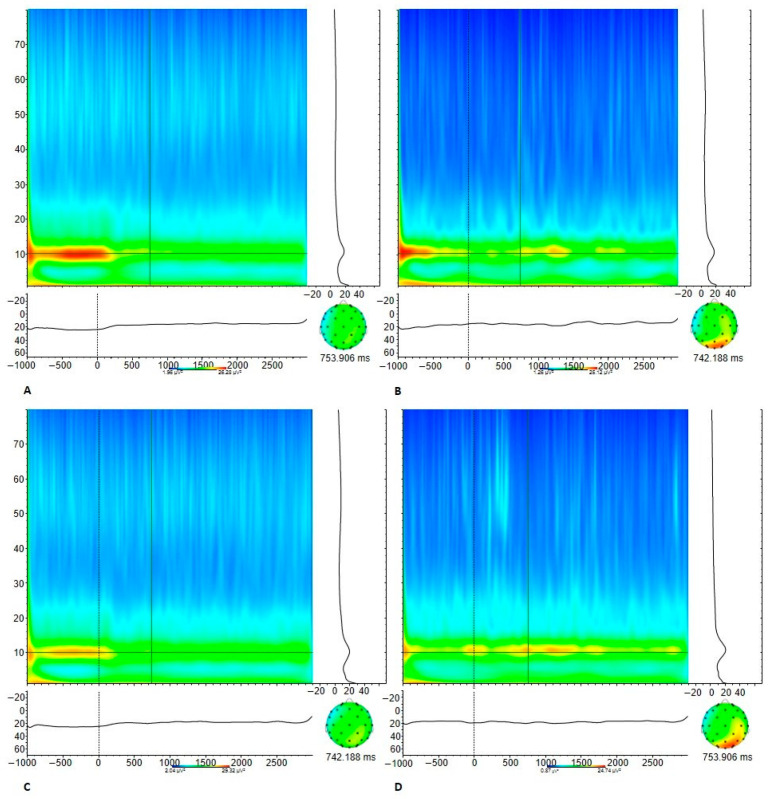
Alpha ERD (at site C4) in aggression condition for (**A**) the delinquent group and. (**B**) controls; and in neutral condition for the (**C**) delinquent group (**D**) controls.

Additionally, the analysis of the alpha power (see Figure 3) showed no significant main effect of condition (*p* = 0.679), nor a main effect for group (*p* = 0.785) and no interaction effect between condition and group (*p* = 0.977).

### 3.4. Regression Analyses

Pooled regression analyses were performed separately for the model predicting reactive aggression and for the model predicting proactive aggression, within the delinquent group (see Table 3).

The overall model predicting reactive aggression, including all six neurobiological measures, was not significant (*p* = 0.929). None of the neurobiological measures significantly contributed to the explanation of reactive aggression.

The overall model predicting proactive aggression, including all six neurobiological measures, was not significant (*p* = 0.920). None of the neurobiological measures significantly contributed to the explanation of proactive aggression.

## 4. Discussion

In this study, we examined differences in psychophysiological and electrophysiological responses between delinquent young adults and controls in a passive viewing task depicting aggressive and neutral interactions. We found one condition effect (significantly higher LPP amplitude while viewing aggressive interactions) and one group effect (higher SCL in the delinquent group compared to controls, irrespective of condition). With respect to the regression analysis, the models including all neurobiological predictors did not significantly predict the variation in reactive and proactive aggression.

The psychophysiological results of the group comparison were partly in line with our expectations. Higher electrodermal activity during an arousal inducing task among delinquent young adults, indicating increased SNS reactivity compared to controls, complies to a certain extent with earlier research [31,32]. Interestingly, the delinquent group not only showed increased SNS reactivity in response to aggressive scenarios, but also towards neutral behaviors. This might indicate an overall hyper-activation of the SNS, which might lead to overwhelmed coping resources and emotion dysregulation. However, we did not find evidence for altered overall ANS reactivity (indicated by HR) or PNS reactivity (indicated by RSA) in response to aggressive and neutral behaviors in the delinquent group, compared to the controls. This does not correspond to some earlier studies but is in line with the mixed results from more recent studies [11,30].

Considering the electrophysiological results, we only found a significant effect of condition for the LPP; however, no significant differences between the groups were found on the P3, LPP and alpha ERD/ mu suppression. The fact that we found a condition effect for only the LPP, and not for the other electrophysiological measures is not in line with prior work using the same task [75,87]. Moreover, the fact that no differences between the groups were found was not in line with our expectations, since earlier studies did find attenuated P3 and LPP amplitudes in affective picture viewing to be related to antisocial personality and delinquency [75,88,89]. Additionally, although studies have found associations between diminished alpha ERD and antisociality [69,70], other studies have found no results concerning alpha ERD (and mu suppression more specifically) and antisocial characteristics [75,88].

Both regression analyses revealed no significant associations between the different neurobiological predictors and reactive aggression and proactive aggression. These outcomes were also not expected, but in line with the results found for our group comparisons.

A possible explanation for these findings might be that HR, RSA, and the P3 are less pure measures of emotional arousal compared to SCL and LPP, since they also respond to attentional processes evoked by a task [32,90]. In that case, our results might probably reflect the characteristics of our sample, demonstrating higher levels of emotional dysregulation and reactive aggression in particular. Although we did not find SCL reactivity to be related to either of the aggression types in this study, heightened SCL has mainly been found to be associated with reactive aggression [34,35].

Furthermore, the results might have been influenced by comorbid addiction problems and the high proportion of non-aggressive offenses (i.e., property crimes) among the delinquent young adults. So, although more than two-thirds of these young adults reported committing an aggressive crime and higher levels of reactive aggression were found in the delinquent group compared to aged-matched controls, our sample could not be considered a severely violent offender group. In a recent meta-analysis, it was shown that psychophysiological effects are largest for the most violent offenders and for psychopathy, and smaller for physical aggression and aggression measured in laboratory tasks [2]. Furthermore, by focusing on indicators of SNS and PNS separately, we might have lost sight of the importance of the cooperation between these two subsystems in understanding emotion dysregulation and aggressive behavior [29,36,43].

Finally, the absence of findings we theoretically expected, might also partly be attributed to the limitations of the current study (see below).

This study has several strengths, such as a clinically relevant and large sample of young delinquents, the combination of multiple neurobiological measures, and the use of a task depicting aggressive behaviors instead of more general stressors to induce arousal.

Despite these strengths, our study also had some important limitations. First, the small sample size of the control group created a power problem for the analyses of group differences and complicated interpretability of the results. However, similar group sizes have been used in other studies on emotional dysregulation [91,92]. Second, we encountered high missingness in the data for both psychophysiological measures (SCL particularly) and electrophysiological measures. In our statistical analysis, we used Multiple Imputation to address this problem. Third, the validity of the task to induce emotional arousal in aggression versus neutral conditions was questionable in the current study. However, previous studies using the same picture task did find support for its validity [75,87]. It may however be that the passive viewing task is less suitable to induce changes, in particular, neurobiological indices such as HR and RSA. Moreover, specific cultural characteristics of the protagonists that were depicted in the scenarios of the pictures (i.e., in all but one of the aggression scenes in which a woman was depicted, a male with a white complexion displayed violence towards a woman with a black skin tone), might have influenced results in this culturally diverse sample. Finally, the use of a self-report questionnaire for aggressive behavior might have distorted the results due to socially desirable response tendencies [93], especially for proactive aggressive behaviors. Since the correlation between reactive and proactive aggression is lower in observation and computer tasks compared to self-report measures [94], operationalization of both constructs should be carefully considered in future aggression research.

## 5. Conclusions

Although differences in psychophysiological and electrophysiological correlates when viewing pictures depicting aggressive interactions (as means to measure emotion regulation) were expected between delinquent young adults and controls, the current study only found an overall increased SCL for the delinquent group. Results also did not support the relation between different neurobiological indices and both reactive and proactive aggression. It is possible that in a (mild) delinquent young adult population, arousal is elevated in general, but not specifically during aggressive situations. However, based on previous literature and theory, it is expected that emotional dysregulation is related to delinquency and other antisocial behaviors, including aggression. In future studies, samples with more severe aggression levels and other types of paradigms and parameters should be considered to elucidate the neurobiological mechanism of emotional dysregulation associated with different types of aggression.

## Figures and Tables

**Table 1:** Demographic variables for delinquent young adults and controls (*N* = 143).

	Delinquent Young Adults (*N* = 118)	Aged-Matched Controls (*N* = 25)
Age	22.54 (2.41)	22.86 (2.55)
Ethnicity		
Western	21 (17.8)	11 (44.0)
Surinamese	23 (19.5)	6 (24.0)
Caribbean	29 (24.6)	2 (8.0)
Moroccan	20 (16.9)	1 (4.0)
Cape Verdean	8 (6.8)	-
Other non-Western	17 (14.4)	5 (20.0)
Education		
Senior secondary education	31 (26.3)	17 (68.0)
Junior secondary education	37 (31.3)	7 (28.0)
Primary education	43 (35.4)	1 (4.0)
None	7 (5.9)	-
Past offenses (official records)		
0	23 (19.5)	-
1–5	55 (46.6)	-
5–10	24 (20.3)	-
>10	16 (13.6)	-
Lifetime delinquency (self-report)		
Destruction/public order offense	79 (67.5)	-
Property offense	98 (85.2)	-
Aggression/violent offense	79 (68.7)	-
Weapon offense	49 (42.6)	-
Drug offense	71 (61.2)	-
Any offense	112 (95.7)	-

Note: Values are presented as mean (SD) for continuous variables or *n* (%) for categorical variables.

**Table 2:** Descriptive statistics and comparative results of behavioral and neurobiological measures (*N* = 143).

	Delinquent Young Adults Mean (SD)	Aged-Matched Controls Mean (SD)	Group Differences *p*-Value
*Behavioral measure*	*(N = 118)*	*(N = 25)*	
Reactive aggression	11.38 (4.51)	8.76 (5.23)	0.026 *
Proactive aggression	5.02 (3.89)	4.00 (4.27)	0.280
Total aggression	16.40 (7.34)	12.76 (9.05)	0.069
*Psychophysiological measures*	*(N = 96)*	*(N = 22)*	
HR-aggressive	65.70 (8.82)	65.75 (8.75)	0.981
HR-neutral	66.44 (8.70)	66.51 (8.63)	0.975
RSA-aggressive	91.24 (34.45)	95.58 (45.28)	0.677
RSA-neutral	95.33 (44.83)	93.40 (41.67)	0.849
SCL-aggressive	5.75 (2.65)	4.08 (2.13)	0.004 **
SCL-neutral	5.78 (2.68)	4.09 (2.13)	0.004 **
*Electrophysiological measures*	*(N = 110)*	*(N = 19)*	
P3-aggressive	4.43 (6.19)	7.63 (4.57)	0.012 *
P3-neutral	4.72 (6.34)	6.25 (4.64)	0.221
LPP-aggressive	5.63 (4.67)	7.66 (4.47)	0.082
LPP-neutral	2.96 (4.70)	3.13 (4.16)	0.868
Mu power-aggressive	16.72 (14.32)	14.68 (9.27)	0.480
Mu power-neutral	17.58 (12.92)	15.90 (10.44)	0.574

Note: The means, SDs and *p*-values reported here are calculated using the original, non-imputed data. Welch’s tests were used to account for the unequal sample sizes. *: significant at α = 0.05; **: significant at α = 0.01.

**Table 3:** Results of pooled regression models predicting reactive and proactive aggression.

Outcome	Predictor	β	SE (β)	*p*	95% CI β	
					Lower	Upper
Reactive aggression	Intercept	11.565	0.661	0.000	10.252	12.879
	Δ HR	0.035	0.287	0.902	−0.535	0.606
	Δ RSA	0.002	0.015	0.909	−0.028	0.031
	Δ SCL	−3.113	3.274	0.346	−9.667	3.443
	Δ P3	0.093	0.134	0.489	−0.174	0.360
	Δ LPP	−0.136	0.136	0.319	−0.405	0.133
	Δ Mu power	0.029	0.084	0.733	−0.140	0.198
Proactive aggression	Intercept	5.432	0.557	0.000	4.325	6.538
	Δ HR	0.083	0.254	0.745	−0.423	0.589
	Δ RSA	−0.003	0.013	0.828	−0.029	0.023
	Δ SCL	−1.239	2.572	0.631	−6.365	3.886
	Δ P3	0.122	0.127	0.337	−0.130	0.375
	Δ LPP	−0.171	0.124	0.170	−0.418	0.075
	Δ Mu power	0.000	0.090	0.996	−0.182	0.183

## Data Availability

The data presented in this study are available on request from the corresponding authors. The data are not publicly available due to the privacy and vulnerability of the young adults participating in our study.
